# Quantifying and reducing inequity in average treatment effect estimation

**DOI:** 10.1186/s12874-023-02104-2

**Published:** 2023-12-15

**Authors:** Kenneth J. Nieser, Amy L. Cochran

**Affiliations:** 1 Department of Population Health Sciences, University of Wisconsin–Madison, Madison, USA; 2 Department of Mathematics, University of Wisconsin–Madison, Madison, USA

**Keywords:** Average treatment effect, Sample representativeness, Subgroup analysis

## Abstract

**Background:**

Across studies of average treatment effects, some population subgroups consistently have lower representation than others which can lead to discrepancies in how well results generalize.

**Methods:**

We develop a framework for quantifying inequity due to systemic disparities in sample representation and a method for mitigation during data analysis. Assuming subgroup treatment effects are exchangeable, an unbiased sample average treatment effect estimator will have lower mean-squared error, on average across studies, for subgroups with less representation when treatment effects vary. We present a method for estimating average treatment effects in representation-adjusted samples which enables subgroups to optimally leverage information from the full sample rather than only their own subgroup’s data. Two approaches for specifying representation adjustment are offered—one minimizes average mean-squared error for each subgroup separately and the other balances minimization of mean-squared error and equal representation. We conduct simulation studies to compare the performance of the proposed estimators to several subgroup-specific estimators.

**Results:**

We find that the proposed estimators generally provide lower mean squared error, particularly for smaller subgroups, relative to the other estimators. As a case study, we apply this method to a subgroup analysis from a published study.

**Conclusions:**

We recommend the use of the proposed estimators to mitigate the impact of disparities in representation, though structural change is ultimately needed.

**Supplementary Information:**

The online version contains supplementary material available at 10.1186/s12874-023-02104-2.

## Background

Historically, racial and ethnic minorities and women have not been afforded the same representation in clinical studies as White men [1, 2]. We refer to the proportion of a sample that belongs to a particular subgroup as that subgroup’s sample *representation*. Despite governmental policies aimed at increasing inclusion of women and racial and ethnic minorities [3–5], reviews of published results from NIH-funded randomized controlled trials have shown that disparities in sample representation persist [6, 7]. Indeed, the NIH RCDC (Research, Condition, Disease Category) Inclusion Statistics report shows large disparities in the typical sample representation of racial and ethnic groups [8]. For example, the median representation of individuals identifying as Asian in cancer studies in 2021 was 2%; less than 1% each for American Indian or Alaska Native, Native Hawaiian or Other Pacific Islander, and individuals indicating more than one race; 8% for Black or African American; and 74% for White; 6% identified as Hispanic or Latino and 87% as not Hispanic or Latino. Disparities in sample representation extend to other segments of the population as well, including older adults [9] and adults with less than 12 years of education [10].

**Fig. 1:**
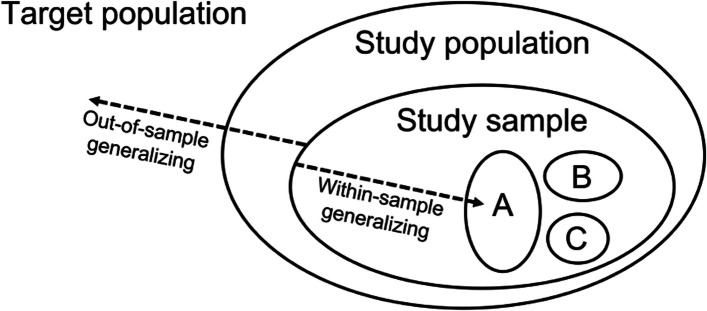
Depiction of two directions of generalization and the relationship between targets of inference. The smallest three ovals represent three mutually exclusive subgroups, labeled A, B, and C, of the study sample

Subgroup sample representation plays a role in the generalizability of average treatment effects (ATEs) in experimental and observational studies. We distinguish two types of generalizability: *out-of-sample* and *within-sample* generalizing (Fig. 1). Researchers seeking to generalize their findings from the sample to a target population, such as a geographic region or a population diagnosed with a particular disease, are generalizing *out-of-sample*. In this setting, if treatment effects vary across subgroups that are disproportionately represented relative to the target population, sample estimates can be biased for the quantity of interest in the target population [11]. Accordingly, researchers might aim to have the representation of subgroups in a sample align with their corresponding representation in a target population. When this is infeasible, analytic methods have been developed for out-of-sample generalizing [12–19].

Even if researchers are able to sample uniformly at random from the target population of interest (or make statistical adjustments to mimic this), results (i.e. average treatment effects) might not generalize equally well across subgroups within the sample. In contrast to out-of-sample generalizing, we refer to generalizing study results to subgroups as *within-sample* generalizing, which is the focus of this paper. Subgroup analyses and hypothesis tests for interactions are common ways to explore and/or confirm within-sample generalizability of ATEs [20, 21]. NIH requires Phase III clinical trials to provide “...valid analysis of whether the variables studied in the trial affect women or members of minority groups...differently than other subjects in the trial...” [5]. Nonetheless, subgroup analyses are not always performed and have been cautioned against for their potentially high statistical noise [22, 23]. When treatment effects vary and when some subgroups systematically have greater representation than others, an ethical question arises: who benefits and who is disadvantaged when researchers generalize a sample ATE to specific subgroups? While intuition might suggest that results will generalize best for subgroups with the greatest sample proportion, we offer a formal approach to answering this question below.

Trading off some bias in estimation for a reduction of noise is one way to ease one of the main concerns with subgroup analysis: imprecision [24, 25]. Biased estimators can borrow strength across subgroups and result in lower mean-squared error, which incorporates both bias and noise, for each subgroup. Bayesian hierarchical modeling provides one route to improving subgroup-specific estimates by borrowing strength across subgroups [24, 26, 27] and these models can be fit using the beanz R package [28]. We distinguish the approach presented in this paper below in the Discussion section.

The key innovation of our approach is to build on existing methodological ideas used for out-of-sample generalizing to the context of within-sample generalizing, by creating pseudo-samples in which representation has been reweighted to improve subgroup effect estimates. This work builds on recent approaches focusing on subgroup-level inferences [29–32]. This approach makes it easy for researchers to (1) use existing unbiased subgroup-specific estimators, (2) combine this method with existing methods for generalizing subgroup-specific effects to a broader target population of interest, and (3) incorporate stakeholder input and judgement into subgroup modeling in a straightforward way. We proceed by first introducing notation used throughout the paper. Under some assumptions, we show how disparities in sample representation affects within-sample generalizability of the sample ATE. We define effects of interest, which we conceptualize as representation-adjusted ATEs, and provide identification proofs and estimators. Lastly, we examine the performance of the proposed estimators in several simulation studies and a case study. We hope that providing a straightforward method for modestly improving the accuracy of subgroup-specific estimates will support researchers conducting subgroup analyses rather than reporting ATEs only.

## Methods

### Notation and definitions

We define the following random variables: $A \in \mathcal {A} = \{0,1\}$ indicates treatment assignment; $X\in \mathcal {X}$ is a vector of baseline covariates; $Y(a) \in \mathbb {R}$ is the potential outcome under assignment to treatment *a*; *Y* is the outcome that is observed; and $S \in \{0,1\}$ indicates sample membership. We observe a sample of *N* independent realizations of $(A, X, Y, S=1)$, where $S=1$ indicates membership in the sample (data not in the study sample would have $S=0$). We assume the data follow a joint distribution $\mathbb {P}$. Since we are interested in subgroups of the sample defined by baseline covariates, we define a partition of $\mathcal {X}$ into *G* mutually exclusive, non-empty subgroups that cover all possible values: $V = \{v_1, \ldots , v_G\}$ That is, each $v_g$ defines a subgroup of individuals with $X \in v_g$ [31].

We define the following quantities: the sample ATE (SATE),

1 $$\begin{aligned} \beta {:=} \mathbb {E}\left[ Y(1) - Y(0)|S=1\right] \, , \end{aligned}$$

the sample representation of individuals with $X \in v_g$,

2 $$\begin{aligned} p_g {:=} \mathbb {P}\left( X \in v_g | S = 1\right) \, , \end{aligned}$$

and the subgroup sample ATE for individuals with $X \in v_g$,

3 $$\begin{aligned} \tau _g {:=} \mathbb {E}\left[ Y(1) - Y(0) | X \in v_g, S=1\right] \, . \end{aligned}$$

We let $p = (p_1, \ldots , p_G)^T$ be the vector of subgroup representation probabilities and $\tau = (\tau _1, \ldots , \tau _G)^T$ be the vector of subgroup sample ATEs. With the law of iterated expectations, $\beta = p^T \tau$. For sake of clarity of exposition, we focus on estimation of the sample ATEs since our focus is on within-sample generalization, but we note that the arguments and methods presented can be generalized to a target population using existing methods [31].

### The impact of disparities in sample representation

Our first task is to reason quantitatively about the impact that disparities in sample representation have on within-sample generalizability. For motivation, we consider a toy example. Researchers conduct five different studies each with three subgroups (groups A, B, and C) with representation fixed to 70%, 20%, and 10% respectively. In each study, researchers obtain an unbiased and very precise estimate of the SATE. We examine how applicable the overall study result (SATE) is for each subgroup by measuring the absolute difference between the SATE and the subgroup’s true effect. Hypothetical data are shown in Table 1.

**Table 1:** Hypothetical data for the toy example comparing the SATE ($\beta$) to subgroup-specific ATEs ($\tau _A, \tau _B, \tau _C$). Bolded values indicate the lowest absolute difference between subgroup-specific effect and SATE in each row

Study	$\tau _A$	$\tau _B$	$\tau _C$	$\beta$	$|\tau _A - \beta |$	$|\tau _B - \beta |$	$|\tau _C - \beta |$
1	-3.51	-2.96	4.04	-2.65	0.87	**0.31**	6.69
2	-4.39	-0.13	4.62	-2.64	**1.75**	2.51	7.26
3	-1.84	-1.08	-0.26	-1.53	**0.31**	0.45	1.27
4	-1.16	-4.71	-4.22	-2.18	**1.02**	2.53	2.05
5	-1.75	0.85	-1.38	-1.19	0.56	2.05	**0.19**

From this example, we see that for studies 2-4, the SATE is closest to the effect of the most represented subgroup (group A). However, in study 1, the SATE is closest to $\tau _B$, and in study 5, the SATE is closest to $\tau _C$. This illustrates that the generalizability of the SATE to a particular subgroup is not solely determined by that subgroup’s representation in the study [33]. The SATE is a weighted average of the subgroup-specific ATEs; although subgroups with greater representation get more weight, an extreme group might pull the average away from a majority group (as in study 1) or two groups with similar effects might benefit from each other’s presence (as in study 5). Consequently, representation is important, but not the only consideration when assessing the within-sample generalizability of results for a given study; knowledge of how much subgroup-specific ATEs are expected to vary is also required.

We formalize these observations by examining the risk function, which specifies the expected loss over repeated samples from the data-generating distribution for a particular estimator and parameter of interest [34]. Under squared error loss, the risk function is the mean squared error (MSE). For an unbiased estimator of the SATE, $\hat{\beta }$, the MSE for estimating $\tau _g$ can be expressed as:

4 $$\begin{aligned} R\left( \tau _g, \hat{\beta }\right) = \sigma ^2_{\hat{\beta }} + (e_g - p)^T\tau \tau ^T(e_g - p) \, , \end{aligned}$$

where $\sigma _{\hat{\beta }}$ is the standard error for $\hat{\beta }$ and $e_g$ is a column vector with the *g*th entry equal to 1 and all other entries equal to 0 (derivation is in the section named "Impact of disparities in representation" of Additional file 1). The risk function (Eq. 4) for $\hat{\beta }$ depends on: 1) the standard error of the SATE estimator and 2) the sample representation of subgroups coupled with the products of all pairs of subgroup ATEs.

Using the risk function directly to draw conclusions about within-sample generalizability is impractical due to its dependence on the unknown subgroup ATEs in $\tau$. To make progress, we propose examining the average risk, also known as the Bayes risk, given a prior distribution or weighting for the parameter values [34]. We model $\tau$ as following a joint distribution $\pi$, which we interpret in two ways. Taking a Bayesian perspective, we can interpret $\pi$ as a prior belief about the probability of different values of $\tau$. Alternatively, from a frequentist perspective, we could interpret $\pi$ to be the distribution of subgroup ATEs that we would observe across many different studies (similar to the toy example above). The latter framing allows us to examine the impact of *systemic* disparities in sample representation.

In general, we define the inequity in average risk between two subgroups, $X \in v_i$ and $X \in v_{j}$ as

5 $$\begin{aligned} \Delta _{i,j}\left( \hat{\theta }_i,\hat{\theta }_{j}\right) {:=} \mathbb {E}_\tau \left[ R\left( \tau _i, \hat{\theta }_i\right) - R\left( \tau _j, \hat{\theta }_j\right) \right] \, , \end{aligned}$$

where $\hat{\theta }_i, \hat{\theta }_j$ are estimators for $\tau _i, \tau _j$, respectively. Critically, inequity in average risk provides us a path forward for reasoning about the impact that disparities in sample representation have on within-sample generalizability. Other aspects of the distribution of differences in MSEs across studies could be considered, but here we focus on the mean difference. As is often the case, when an unbiased SATE estimator is used to obtain a single estimate from a sample, the inequity for individuals with $X \in v_1$ relative to individuals with $X \in v_2$ is

6 $$\begin{aligned} \Delta _{1,2} \left( \hat{\beta }, \hat{\beta }\right) = \text {tr}\left\{ \left( (e_1-p)(e_1 - p)^T - (e_2-p)(e_2 - p)^T\right) \mathbb {E}_\tau \left[ \tau \tau ^T\right] \right\} \, . \end{aligned}$$

To calculate the inequity measure of $\hat{\beta }$ in Eq. 6, we do not need to specify the full distribution of $\tau$ but rather only $\mathbb {E}_\tau [\tau \tau ^T] = \Sigma _\tau + \mu _\tau \mu _\tau ^T$, where $\Sigma _\tau$ and $\mu _\tau$ are the covariance matrix and mean vector for $\tau$, respectively. If researchers are uncertain how the treatment effect varies across subgroups, we recommend they assume $\pi$ is exchangeable across subgroups (i.e. permutation of subgroup labels leaves the joint distribution $\pi$ unchanged) [27]. This results in the following simplification,

7 $$\begin{aligned} \Delta _{1,2} \left( \hat{\beta }, \hat{\beta }\right) = \phi ^2(p_2 - p_1) \, , \end{aligned}$$

where $\phi ^2 = \text {var}_\tau (\tau _i - \tau _j) = \mathbb {E}_\tau [(\tau _i - \tau _j)^2]$ for any $i \ne j$.

Equation 7 has consequential implications: when treatment effects vary and researchers report only average treatment effects, results will be less applicable on average for subgroups with lower representation. This holds both in a single study in which researchers have no prior knowledge of subgroup effect heterogeneity and across a collection of studies with consistent representation disparities. The inequity in average risk between two subgroups is directly proportional to the difference in representation of the subgroups and the variance of subgroup-specific ATE differences, under the assumptions given above. At the design stage of a study, given an approximation of how much treatment effects are expected to vary across subgroups $\phi$, this simple formula could inform how much disparity in representation could be tolerated during study enrollment. At the analysis and interpretation stage, researchers can use Eq. 7 to more quantitatively reason about the within-sample generalizability of study results, by considering both disparities in representation and expectations of subgroup differences in ATEs.

In cases where treatment effects are expected to vary substantially across subgroups, researchers could sample subgroups in equal proportion, so that $p_2 - p_1 = 0$, to eliminate the inequity in Eq. 7. However, there are no guarantees on how accurate the SATE estimate would be for each of the subgroups. Another option is to change the estimator used to obtain each of the subgroup-specific estimates. Simple alternatives would be to obtain unbiased estimates of subgroup-specific treatment effects by stratifying the analysis by subgroup or fitting a regression model of the outcome with treatment-subgroup interaction terms. We quantify the inequity of this approach as

8 $$\begin{aligned} \Delta _{1,2} \left( \hat{\tau }_1, \hat{\tau }_2\right) = \mathbb {E}_\tau \left[ \sigma _1^2 - \sigma _2^2\right] \, , \end{aligned}$$

where $\hat{\tau }_1$, $\hat{\tau }_2$ are unbiased subgroup-specific estimators of $\tau _1, \tau _2$, respectively, and $\sigma _1, \sigma _2$ are the corresponding standard errors. In large samples, $\sigma _1$ and $\sigma _2$ will tend to be small. In small samples, when $p_2 > p_1$, we generally have that $\sigma _2 < \sigma _1$ due to having fewer data for the less represented group. This implies that subgroups with less representation will have higher risk on average. While this approach addresses the inequity due to differences in the bias of the SATE estimator, it creates another issue due to differences in variance. Next, we discuss a third option which seeks to find a balance between these two alternatives.

### Representation-adjusted ATEs

When estimating a subgroup-specific ATE, we need not completely dispense with the information provided by the other subgroups in the sample. Effect sizes for some subgroups in the sample can give an approximate sense of reasonable values for the effect sizes of other subgroups. For example, if we know that for many subgroups, the ATE is generally an increase of the outcome by 2 to 5 units, then a subgroup-specific effect of 20 units would be suspect (though not impossible). When subgroups are analyzed separately, this valuable information is lost. Similar to Bayesian analyses of subgroup effects [25, 27, 35], we sought to make use of this information to improve the precision of subgroup-specific ATE estimation.

Since we noted that differences in representation led to inequity in average MSE, we consider multiple pseudo-samples in which all sample data are retained for each subgroup. In each, subgroup representations are adjusted in an optimal way to improve the accuracy of the SATE for the subgroup of interest. We develop a method that does not require specification of full prior distributions and is less computationally expensive than fully Bayesian approaches. In addition, our approach allows for correlated subgroup-specific ATE estimators. We denote membership in the representation-adjusted sample for individuals with $X \in v_g$ with the discrete random variables $S_g$, taking values 0 or 1. The observed sample indicator is still denoted by *S* without a subscript. For each $g = 1,\ldots , G$, we define new effects of interest as

9 $$\begin{aligned} \eta _{g} {:=} \mathbb {E}\left[ Y(1) - Y(0) | S_g = 1\right] \, . \end{aligned}$$

We refer to this effect as a representation-adjusted ATE (RATE) for individuals with $X \in v_g$. The degree of representation adjustment will depend on both the amount of information we have for each subgroup and prior expectations of how much subgroup ATEs differ, which we discuss under *Estimation and inference* below.

#### Identification

The RATE can be expressed as a function of observed variables $(A,X,Y,S=1)$ under certain assumptions. We assume that mean potential outcomes in the subgroup in the observed sample are equal to mean potential outcomes in the same subgroup in the pseudo-sample; that is, $\mathbb {E}[Y(a) | X \in v_g, S=1] = \mathbb {E}[Y(a) | X \in v_g, S_g = 1]$, for all *g* and $a \in \mathcal {A}$. With this assumption of exchangeability over sample indicators—which is different than the subgroup effect exchangeability assumption we discussed in the previous subsection—the RATE can be expressed as a weighted average of the subgroup-specific effects as follows,

10 $$\begin{aligned} \eta _{g}&= \mathbb {E}[Y(1) - Y(0)|S_g=1]\nonumber \\&= \sum _{k=1}^G \mathbb {P}(X \in v_k|S_g=1)\mathbb {E}[Y(1) - Y(0)|X \in v_k, S_g=1]\nonumber \\&= \sum _{k=1}^G \mathbb {P}(X \in v_k|S_g=1)\mathbb {E}[Y(1) - Y(0)| X \in v_k, S=1] \nonumber \\&= \sum _{k=1}^G \mathbb {P}(X \in v_k|S_g=1)\tau _k \, , \end{aligned}$$

where the first equality is the definition of the effect, the second follows from the law of iterated expectations, the third follows from the assumption of exchangeability over sampling indicators, and the last follows from the definition of $\tau _k$. Equation 10 is analogous to the transport formula given in [14]. Note that the equalities shown above could be applicable to both experimental and observational studies. In the case of observational studies, however, assumptions about exchangeability of treatment assignment are necessary for the identification of $\tau _k$. Steps for identifying the subgroup-specific effects $\tau _k$ closely follow the identification proofs in [31] which we detail in the section named "Identification" of Additional file 1. If out-of-sample generalizing is of interest, $\tau _k$ can simply be replaced with the respective subgroup effects in the target population. Again, identification arguments follow those in [31] as described in the section named "Identification" of Additional file 1.

#### Estimation and inference

Based on Eq. 10, RATE estimators can be expressed as:

11 $$\begin{aligned} \tilde{\tau }= Q \hat{\tau }\, , \end{aligned}$$

where $\tilde{\tau }$ is a vector of RATE estimators, *Q* is matrix of probabilities with entries $q_{ij} = \mathbb {P}(X \in v_j | S_i = 1)$, and $\hat{\tau }$ is a vector of unbiased estimators for $\tau$. Accordingly, we need to 1) unbiasedly estimate the subgroup-specific ATEs: $\hat{\tau }$ and 2) specify subgroup probabilities in the representation-adjusted samples *Q* which we treat as fixed. For step 1, the estimators presented in [31] can be adapted to estimate sample-specific subgroup effects by conditioning on sample membership. For example, a typical outcome modeling approach might estimate the subgroup-specific ATEs as

12 $$\begin{aligned} \hat{\tau }= \mathbb {E}_n\left[ \hat{g}_1(X) | X \in v_g, S=1\right] - \mathbb {E}_n\left[ \hat{g}_0(X) | X \in v_g, S= 1\right] \, , \end{aligned}$$

where $\mathbb {E}_n[\cdot ]$ denotes an empirical expectation and $\hat{g}_a(X)$ is an estimator for $\mathbb {E}[Y|X, S=1, A = a]$ for $a = 0, 1$. Inverse-probability weighting estimators, augmented or not, are another possibility.

For step 2, we consider two approaches. First, we can use estimated probabilities that minimize the average MSE for each subgroup. This approach is motivated by the notion that what is fair is to provide each subgroup with the best estimator possible given the data, where the best estimator is defined by minimal average MSE. In general, this requires specification of a particular prior distribution for the subgroup effects, but we partially avoid this by assuming exchangeability across subgroups. Assuming exchangeability, minimizing the average MSE yields the following construction for representation-adjusted samples for subgroup *g*, which we refer to as the optimal weights (detailed derivations in the section named "Specifying subgroup representation for RATE estimators" of Additional file 1):

13 $$\begin{aligned} q_g^{\text {optimal}} = \left( 1 - \textbf{1}^T \Omega e_g\right) \left( \textbf{1}^T \Omega \textbf{1}\right) ^{-1} \Omega \textbf{1} + \Omega e_g \, , \end{aligned}$$

where $q_g^T$ is the *g*th row of *Q*, $\Omega = \phi ^2 (2\Sigma _{\hat{\tau }} + \phi ^2 \mathbb {I})^{-1}$, $\Sigma _{\hat{\tau }}$ is the covariance matrix for $\hat{\tau }$, and $\phi ^2$ was defined in the last section as $\text {var}_\tau (\tau _i - \tau _j)$ for $i\ne j$. When the set of subgroup-specific effects $\tau$ are uncorrelated and the set of subgroup-specific estimators $\hat{\tau }$ are uncorrelated, the RATE estimator corresponds to the pooled estimator from a simple Bayesian normal hierarchical model with a uniform prior on the hypermean and fixed hypervariance for $\tau$.

One potential concern might be that the optimal weights could still result in disparities in the representation of each subgroup within their respective representation-adjusted sample, with larger subgroups tending to have greater representation. To address this, a second way to specify probabilities in *Q* could follow a similar process but constrain subgroup probabilities to be the same for each representation-adjusted sample. Adding constraints to optimization algorithms has been a common way of tackling unfairness in model performance in other applications [36, 37]. One way to force representation to be the same for the effect estimation for each subgroup is to require $q_g = w e_g + (G-1)^{-1}(1-w)(\textbf{1} - e_g)$ for some $w \in [0,1]$. Then, we can choose a *w* to use for all subgroups by minimizing a joint function of the subgroup-specific average MSEs—specifically we consider the average MSE averaged over the subgroups. Under mild regularity conditions, this yields the following representation probabilities for the RATE for subgroup *g*, which we refer to as the shared weights (details in the section named "Specifying subgroup representation for RATE estimators" of Additional file 1):

14 $$\begin{aligned} q_g^{\text {shared}} = \frac{1}{1 + \gamma }e_g + \frac{\gamma }{1 + \gamma }\frac{\textbf{1}-e_g}{G-1} \, , \end{aligned}$$

where $$\gamma = \frac{\bar{\sigma ^2}(G-1) - V_1}{\phi ^2G/2 + V_2/(G-1) - V_1}$$, $$\bar{\sigma ^2} = G^{-1}\sum _{g\in \mathcal {G}}\sigma _g^2$$, $$\sigma _g^2 = \text {var}(\hat{\tau }_g)$$, $$V_1 = G^{-1}\sum _{g\in \mathcal {G}} e_g^T\Sigma _{\hat{\tau }}(\textbf{1} - e_g)$$, and $$V_2 = G^{-1}\sum _{g\in \mathcal {G}} = (\textbf{1}-e_g)^T\Sigma _{\hat{\tau }}(\textbf{1}-e_g)$$.

**Fig. 2:**
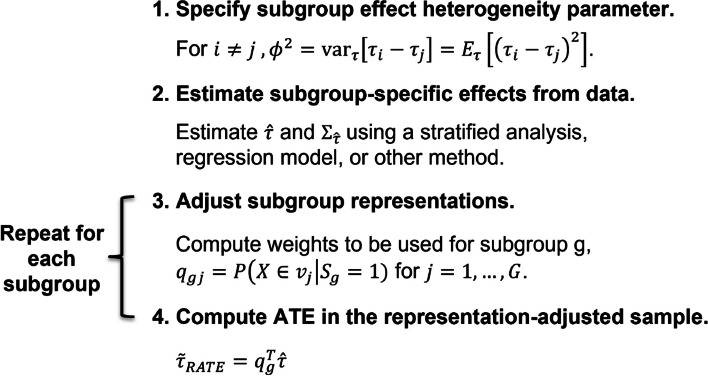
Steps to estimating RATEs

In practice, using either approach, researchers could specify $\phi$ or a range of $\phi$ values directly based on substantive knowledge and empirically estimate the values of $\hat{\tau }$ and $\Sigma _{\hat{\tau }}$. Note in Eqs. 13 and 14 that as $\phi \rightarrow \infty$, $q_g \rightarrow e_g$. This means that for large values of $\phi$, subgroups are effectively analyzed separately and consequently, estimates become unbiased. In other words, large values of $\phi$ effectively stratify the analysis by subgroup. On the other extreme, setting $\phi = 0$ effectively ignores possible treatment effect heterogeneity. Choosing an intermediate value of $\phi$, based on an expectation of the amount of treatment effect heterogeneity, permits some bias for a reduction in variance. To help reason about appropriate values for $\phi$, researchers could make use of Popoviciu’s inequality on variances [38] which implies that if the difference in subgroup-specific ATEs is bounded, that is $\mathbb {P}(|\tau _i - \tau _j| \le c) = 1$, then $\phi = \text {SD}[\tau _i - \tau _j] \le c/2$. If a researcher knows that subgroup-specific ATEs should not differ by more than 2 units, then $\phi$ should be no more than 1. The Bhatia-Davis inequality is another option [39]. Figure 2 summarizes the steps a researcher would take to estimate RATEs. Note that $\Sigma _{\hat{\tau }}$ in Eqs. 13 and 14 needs to be replaced with with an estimate $\hat{\Sigma }_{\hat{\tau }}$.

Representation-adjusted samples constructed in this way have some useful statistical properties. As the sample size grows, standard errors for subgroup estimators will shrink causing the representation for the subgroup of interest to approach 1. This means that RATE estimators are asymptotically unbiased (further detail in the section named "Large sample properties of RATE" of Additional file 1). As a result, improvements in the recruitment of participants from a subgroup naturally will reduce the bias in estimation, but in contexts where this is infeasible, this approach enables small subgroups to wield the full information of the data sample to inform their estimate. Lastly, inference for the RATE estimator is straightforward given it is a weighted average of the subgroup-specific estimators ($\hat{\tau }$). The covariance matrix of the RATE estimators is given by $\Sigma _{\tilde{\tau }} = Q\Sigma _{\hat{\tau }}Q^T$, which could be used to construct confidence intervals. Non-parametric bootstrap intervals are another option.

### Simulation

While the optimally weighted RATE estimator yields the lowest average MSE across a broad class of estimators, the distribution of MSE, sensitivity to misspecifying $\phi$, and impact of adding a shared weight constraint is unclear. To address these questions, we simulated randomized controlled trials of a binary treatment and continuous outcome with sample size of 300. For simplicity, we assumed that trial participants were sampled uniformly at random from some target population. We considered trials with either three or five subgroups, and trials with subgroup-specific effects independently and identically drawn from three different distributions (standard normal; bimodal; Gamma(3,3)) for a total of six different scenarios. The bimodal distribution was a mixture of two normal distributions: N(0.5, 1) with probability 0.8 and N(-3, .5) with probability 0.2. All distributions were scaled such that the true value of $\phi$ was 1. In trials with three subgroups, representation was fixed to 75%, 15%, and 10% for groups 1-3 respectively. For trials with five subgroups, representation was fixed to 67%, 15%, 10%, 5%, 3% for groups 1-5 respectively. Treatment was randomly allocated with equal proportions within each subgroup to ensure treatment balance. Outcomes were generated as follows:

15 $$\begin{aligned} Y = 1 + \beta _G A + 2 \mathbbm{1}_{\{G = 1 \text { or } 3\}} + X_1 + 2.5 X_2 + \epsilon , \end{aligned}$$

where *A* is a binary indicator of treatment assignment, $\beta _G$ are the sampled subgroup effects for group *G*, $X_1$ is a continuous covariate sampled from *N*(1, 1), $X_2$ is a binary covariate sampled from a Bernoulli(0.3), and $\epsilon$ is random error sampled from *N*(0, 1).

For each of the six scenarios, we drew 500 sets of subgroup effects and for each set, we simulated 500 data samples from which we estimated the root mean squared error (RMSE) of subgroup-specific effect estimates. In each data sample, we obtained estimates from a stratified model, regression model with treatment-subgroup interaction terms, a model with a random effect for the treatment, and four different RATE estimators. Subgroup-specific effect estimates from the regression model were obtained using the multcomp R package [40]. In the random effects model, we obtained subgroup-specific predicted effects using the lme4 R package [41]. The random effects estimator is equivalent to a basic Bayesian shrinkage estimator with a fixed value for the prior variance of the subgroup-specific effects and non-informative prior for the mean of the subgroup-specific effects [25]; this served as an easy-to-implement substitute for comparing the RATE estimators to a fully Bayesian hierarchical model. The RATE estimators used subgroup-specific estimates from the interaction model. For three of the RATE estimators, we used the optimal weights with different values of $\phi$ (0.75, 1, 1.5); these values were chosen to undervalue, appropriately value, and overvalue the true value of $\phi$, respectively. For the fourth RATE estimator we used the shared weights with $\phi = 1$. We plotted cumulative estimates of the 25th, 50th, and 75th percentile of the RMSE distribution to confirm that estimates had stabilized after 500 draws from the subgroup effect distribution. All simulations were performed in R Statistical Software v4.2.1 [42].

## Results

### Simulation results

**Fig. 3:**
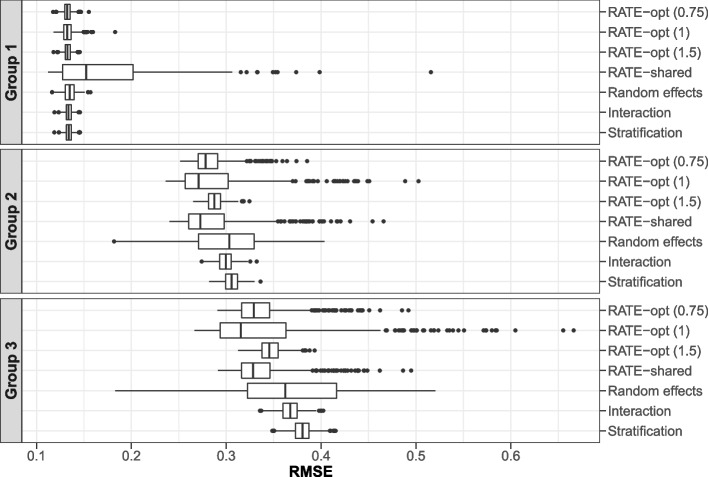
Box plots of estimated RMSE of subgroup-specific treatment effect estimators from simulation of randomized controlled trials with binary treatment, continuous outcome, and $N = 300$. Results are based on 500 draws of 3 subgroup effects from a standard normal distribution scaled so that $\phi ^2 = 1$

In the scenario with three subgroup effects drawn from a common normal distribution, we found that all the RATE estimators had slightly lower median RMSE than the other estimators for groups 2 and 3, even when $\phi$ was misspecified. However, in cases where a given subgroup’s true effect happened to be very different from the effect in the other subgroups, the RMSE from the RATE estimators for the given subgroup was high. Boxplots of estimated RMSE of the subgroup-specific effect estimators are shown in Fig. 3; corresponding summary statistics are shown in Additional file 1 (Table S1). RATE estimators with a shared set of weights had similar performance to those with optimal weights for groups 2 and 3, but resulted in substantially worse performance for group 1, the largest group. Except for the shared weight RATE estimator, other estimators had similar RMSE for group 1. There was considerably more variability in the RMSE from the random effects model estimates for groups 2 and 3 compared to the other estimates. Corresponding figures for the five remaining scenarios are shown in Additional file 1 (Figs. S1–S5); results were generally consistent.

### Empirical example

To demonstrate the use of this method in an applied example, we estimated RATEs using results from an analysis of the Moving to Opportunity study (MTO) [43]. The MTO ran from 1994 to 1998 and was sponsored by the U.S. Department of Housing and Urban Development in five U.S. cities. Briefly, the MTO randomly assigned families living in public housing in high-poverty areas to receive a voucher that would subsidize rent in the private market. The authors in [43] assessed how rental subsidies impacted psychological distress and behavioral problems of the children in the study, focusing on effect modification by gender and family health vulnerability. Families were considered “vulnerable” if any household member had a disability or any child in the household had a health or developmental problem. We focus on the analysis of psychological distress which was measured using standardized factor scores from a latent variable analysis of the Kessler 6 scale.

The authors in [43] found that the intervention benefited girls from nonvulnerable families but had a detrimental effect on boys from vulnerable families. We explored how robust these findings were when there was little prior expectation of these differences. We used published estimates for each of the four groups from [43]—nonvulnerable girls, vulnerable girls, nonvulnerable boys, vulnerable boys—and calculated corresponding standard errors based on the 95% confidence intervals. Although these estimates are likely correlated since they are not from a fully stratified model, we assumed they were uncorrelated for illustrative purposes. We assumed that subgroup-specific effects should not differ by more than 0.25 SDs. Based on Popoviciu’s inequality on variances, we would expect that $\phi$, the standard deviation of differences in subgroup-specific effects, is less than or equal to 0.125. With $\phi = 0.125$, we obtained RATE representation, estimates, and 95% confidence intervals (Table 2). The full *Q* matrix can be found in the section named "Case study detail" of the Supplementary Material. Even under this skeptical prior, there is still evidence of a difference in treatment effects between groups. Additionally, RATE estimates provide tighter confidence intervals for all subgroups compared to the original subgroup-specific estimates.

**Table 2:** NVG: nonvulnerable girls, VG: vulnerable girls, NVB: nonvulnerable boys, VB: vulnerable boys. Stratified and RATE representation and estimates of the impact of MTO on psychological distress. RATE estimates were calculated using $\phi = 0.125$

Group	Sample representation	Original estimates	RATE-opt representation	RATE-opt estimates
NVG	30.9%	-0.21 (-0.34 to -0.07)	72.4%	-0.12 (-0.22 to -0.02)
VG	19.5%	0.02 (-0.15 to 0.18)	63.3%	0.02 (-0.09 to 0.12)
NVB	26.9%	0.04 (-0.09 to 0.17)	73.2%	0.03 (-0.07 to 0.13)
VB	22.7%	0.26 (0.09 to 0.44)	60.5%	0.14 (0.03 to 0.25)

## Discussion

Some population subgroups—including, but not limited to, racially and ethnically marginalized groups, women, and older adults—consistently have lower representation in experimental and observational studies compared to their counterparts. In many cases, such as investigations of associations between Framingham risk factors and cardiovascular disease [44] and genetic studies of various health outcomes [45], imbalance in study representation has led to study findings that generalize better for subgroups with greater representation. In other cases, such as randomized controlled trials related to depression, the impact of this imbalance is unknown because the heterogeneity in treatment effects is left unexplored [46]. In this paper, we have presented a statistical framework for understanding this phenomenon, focusing on SATE estimation, that can partially inform the design, analysis, and interpretation of studies in heterogeneous populations. We showed that the difference between subgroups in average risk (MSE) of the SATE estimator increases linearly with the disparity in representation and with the variance of treatment effect differences. Improving data collection and community engagement will be essential to addressing the inadequate inclusion of marginalized groups in experimental and observational studies and reduce this inequity.

In practice, given that many studies do have substantial disparities in representation, we sought to improve estimation accuracy, on average across studies. Motivated by the idea that sample representation has an impact on the generalizability of study results and that changing or adjusting sample representation for less represented subgroups could improve generalizability for these groups, we introduced a new effect of interest which we refer to as a RATE, which is the ATE in a representation-adjusted sample. In general, the RATE is any weighted average of subgroup-specific effects. The RATE estimators require researchers to input into the analysis how different they expect subgroup-specific effects to be. Estimating the SATE or unbiased subgroup-specific effects are particular cases of the RATE estimators in which researchers either implicitly assume that subgroup-specific effects are completely homogeneous or completely distinct from one another, respectively. Specification of $\phi$ allows for a balance between these choices and could be a discussion among key stakeholders and community members of the relevant sociodemographic subgroups. After $\phi$ is specified, the unbiased subgroup-specific effects are optimally weighted and combined in a different way for each subgroup to minimize the average risk. This method can improve upon simple unbiased subgroup-specific estimates by borrowing strength from the other subgroups. With that said, the performance of the RATE estimator in any given study will depend on the true, unknown subgroup-specific effects in that study. The theoretical results presented in this paper show that the RATE estimator can provide the lowest MSE on average across many studies.

We explored an alternative RATE estimator in which subgroups were constrained to use a shared set of representation probabilities. We found in our simulations that this led to substantially worse performance for the largest subgroup relative to the optimally weighted RATE. While a set of shared weights might be valued for its ability to give subgroups equal representation, lower average MSE can always be achieved by using the optimally weighted RATE. In many scenarios in which algorithmic fairness is a concern (e.g., employment, criminal sentencing, and loan applications), relevant parties generally differ in their goals (e.g. hiring the best candidates vs. securing a job) leading to deliberation of what is or is not fair. Aside from cases of scarce resource allocation (e.g., organ transplantation), medical care differs in that all parties share the same goal: improving patient health [47]. Consequently, obtaining the most accurate estimates of treatment effectiveness possible for each subgroup should be the main objective. For this reason, we view the optimally weighted RATE as a more equitable approach to adjusting sample representation.

We distinguish the RATE estimators from Bayesian subgroup modeling, such as the methods discussed in [27], in a few ways. First, we do not make assumptions about the exact distribution of the underlying subgroup effects and relevant hyperparameters as a fully Bayesian approach would. Second, we allow for correlation between the subgroup-specific effect estimators. Subgroup-specific estimators obtained from a regression model adjusted for covariates are typically correlated unless covariate effects are estimated separately for each subgroup. This correlation allows for additional information-borrowing. Third, we specify the standard deviation of the differences in subgroup effects directly rather than incorporate an estimate from the data. Estimating this parameter from the data can be challenging when the number of subgroups is small. In simulation, we saw that in the case of 3 subgroups, the random effects estimator performed substantially worse. However, there were some cases in which specifying this parameter directly led to large RMSEs as well.

The RATE estimator with optimal weights relies on the assumption that the subgroup effects are exchangeable, which is typical for Bayesian subgroup analyses though more complex methods are available [27]. The full exchangeability assumption could be weakened and would require additional hyperparameters to be specified by the researcher based on the number of subsets of subgroup effects that reasonably would be exchangeable. Future studies could explore this extension.

Finally, one of the complications of studying effect modification is that it is scale-dependent. Comparisons of subgroup-specific ATEs on the difference scale might be smaller, larger, or non-existent than comparisons on the risk ratio and/or odds ratio scales. In fact, if baseline risk of an outcome varies across subgroups, then treatment effectiveness will vary on at least one scale. In this article, we focused on the risk difference scale, which is easy to interpret as well as the scale with greatest public health and policy importance [48, 49]. The approach we presented can be extended to other scales by shifting focus to estimating the potential outcome means under different treatment assignment and then combining them in the appropriate way. Identification and estimator derivation logic would remain the same but lead to the need to specify two hyperparameters—the standard deviation of mean difference under treatment and the standard deviation of mean differences under control—rather than just one—the standard deviation of treatment effect differences.

## Conclusions

In conclusion, the framework laid out in this article provides a way to quantitatively assess the impact of reporting only the SATE when there is disparity in representation across population subgroups. Estimators that borrow strength across subgroups, such as the RATE estimator, can reduce the inequitable impact at the data analysis stage. Ultimately, structural change regarding data collection and funding priorities is needed to address systemic disparities in sample representation [50].

### Supplementary Information

**Additional file 1.** Supplementary material. This file contains additional mathematical details and support accompanying the main text, along with figures with results from the rest of the simulation studies and case study details.

## Data Availability

Source code for simulations and RATE estimation are publicly available on Github at https://github.com/knieser/RATE.

## Abbreviations

ATE: Average treatment effect
SATE: Sample average treatment effect
RATE: Representation-adjusted average treatment effect
SD: Standard deviation
MSE: Mean-squared error
RMSE: Root mean-squared error
