# Supplementary material for: Quantifying and reducing inequity in average treatment effect estimation
Source: BMC Med Res Methodol. 2023 Dec 15;23:297. doi: 10.1186/s12874-023-02104-2 (PMC10722685; doi:10.1186/s12874-023-02104-2)
Supplement: Supplementary file 1 — Additional file 1. Supplementary material. This file contains additional mathematical details and support accompanying the main text, along with figures with results from the rest of the simulation studies and case study details. [file 12874_2023_2104_MOESM1_ESM.pdf]

Additional file 1 – Supplementary material

This file contains additional mathematical details and support accompanying the main text, along with figures with results from the rest of the simulation studies and case study details.

Impact of disparities in representation

Let $p = (p_1, \dots, p_G)^T$ be the group probabilities in the sample (i.e. $p_g = \mathbb{P}(X \in v_g | S = 1)$), $\tau = (\tau_1, \dots, \tau_G)^T$ be the subgroup ATEs, and $\beta = p^T\tau$ be the SATE. Let $\hat{\beta}$ be an unbiased estimator for the SATE. We are interested in the risk of using $\hat{\beta}$ as an estimator for the subgroup ATEs. In other words, for each $g \in 1, \dots, G$, we are interested in the risk function:

$$R(\tau_g, \hat{\beta}) \coloneqq \mathbb{E}\left[\left(\tau_g - \hat{\beta}\right)^2 \Big| \tau\right] .$$

with expectation taken over $P(A, X, Y, S)$ keeping τ fixed. We can express the risk function as:

$$\begin{aligned}
R(\tau_g, \hat{\beta}) &= \mathbb{E}\left[\left(\tau_g - \hat{\beta}\right)^2 \Big| \tau\right] \\
&= \mathrm{var}\left[\tau_g - \hat{\beta}\Big|\tau\right] + \left(\mathbb{E}[\tau_g - \hat{\beta}|\tau]\right)^2 \\
&= \mathrm{var}\left[\hat{\beta}\Big|\tau\right] + (\tau_g - \beta)^2 \\
&= \mathrm{var}\left[\hat{\beta}\Big|\tau\right] + \left(\tau_g - p^T\tau\right)^2 \\
&= \mathrm{var}\left[\hat{\beta}\Big|\tau\right] + \left((e_g - p)^T\tau\right)^2 \\
&= \sigma^2_{\hat{\beta}} + (e_g - p)^T\tau\tau^T(e_g - p) ,
\end{aligned}$$

where the third equality follows from the unbiasedness of $\hat{\beta}$, $\sigma_{\hat{\beta}} := \sigma_{\hat{\beta}}(\tau)$ is the standard error for $\hat{\beta}$, and e_g is a column vector where the gth entry is 1 and all other entries are 0.

Under the average risk perspective, we assume that the subgroup ATEs, τ, follow a joint distribution π with mean μ_τ and covariance matrix Σ_τ. We are interested in the expectation over π,

$$\mathbb{E}_\tau[R(\tau_g, \hat{\beta})] = \mathbb{E}_\tau\left[\sigma^2_{\hat{\beta}}\right] + (e_g - p)^T\,\mathbb{E}_\tau[\tau\tau^T](e_g - p) .$$

The inequity in average risk using the sample ATE to estimate τ_1 relative to τ_2 can be expressed as

$$\begin{aligned}
\mathbb{E}_\tau[R(\tau_1, \hat{\beta})] - \mathbb{E}_\tau[R(\tau_2, \hat{\beta})] &= (e_1 - p)^T\,\mathbb{E}_\tau[\tau\tau^T](e_1 - p) - (e_2 - p)^T\,\mathbb{E}_\tau[\tau\tau^T](e_2 - p) \\
&= \mathrm{tr}\left\{\left((e_1 - p)(e_1 - p)^T - (e_2 - p)(e_2 - p)^T\right)\mathbb{E}_\tau[\tau\tau^T]\right\} .
\end{aligned}$$

Now suppose that π is invariant under permutations of the subgroup labels (i.e. $(\tau_1, \dots, \tau_G)$ is an exchangeable set of random variables). Then we have

$$\begin{aligned}
\mathbb{E}[\tau] &= \mu_\tau\mathbf{1} \\
\Sigma_\tau &= \sigma^2_\tau\left((1 - \rho)I + \rho\mathbf{1}\mathbf{1}^T\right) ,
\end{aligned}$$

where $\sigma^2_\tau = \mathrm{var}_\tau[\tau_g]$ for all g, and $\rho = \mathrm{cov}(\tau_i, \tau_j)/\sigma^2_\tau$ for all $i \neq j$. With this, we can simplify the expression as follows:

$$\begin{aligned}
\mathbb{E}_\tau[R(\tau_1, \hat{\beta})] - \mathbb{E}_\tau[R(\tau_2, \hat{\beta})] &= \mathrm{tr}\left\{\left((e_1 - p)(e_1 - p)^T - (e_2 - p)(e_2 - p)^T\right)\left(\sigma^2_\tau(1 - \rho)I + \sigma^2_\tau\rho\mathbf{1}\mathbf{1}^T + \mu^2\mathbf{1}\mathbf{1}^T\right)\right\} \\
&= \sigma^2_\tau(1 - \rho)\mathrm{tr}\left\{(e_1 - p)(e_1 - p)^T - (e_2 - p)(e_2 - p)^T\right\} \\
&= \sigma^2_\tau(1 - \rho)\left((1 - p_1)^2 + p_2^2 - p_1^2 - (1 - p_2)^2\right) \\
&= 2\sigma^2_\tau(1 - \rho)(p_2 - p_1) \\
&= \phi^2(p_2 - p_1) ,
\end{aligned}$$

where $\phi = \mathrm{SD}[\tau_i - \tau_j]$ for $i \neq j$, since

$$\mathrm{tr}\left\{(e_k - p)(e_k - p)^T \mathbf{1}\mathbf{1}^T\right\} = \left((e_k - p)^T \mathbf{1}\right)^2 = 0 \qquad k = 1, 2.$$

This implies that when $p_2 > p_1$, the average risk using the sample ATE to estimate τ_1 is higher than the average risk using the sample ATE to estimate τ_2.

Now let us consider a stratified analysis. For individuals with $X \in v_1$, we use an unbiased estimator $\hat{\tau}_1$ of τ_1.

$$\begin{aligned} R(\tau_1, \hat{\tau}_1) &= \mathbb{E}\left[(\tau_1 - \hat{\tau}_1)^2 \middle| \tau\right] \\ &= \mathrm{var}\left[\tau_1 - \hat{\tau}_1 \middle| \tau\right] \\ &= \mathrm{var}\left[\hat{\tau}_1 \middle| \tau\right]. \end{aligned}$$

Then

$$\mathbb{E}_\tau\left[R(\tau_1, \hat{\tau}_1)\right] = \mathbb{E}_\tau\left[\mathrm{var}\left[\hat{\tau}_1 \middle| \tau\right]\right]$$

and

$$\mathbb{E}_\tau[R(\tau_1, \hat{\tau}_1)] - \mathbb{E}_\tau[R(\tau_2, \hat{\tau}_2)] = \mathbb{E}_\tau\left[\mathrm{var}\left[\hat{\tau}_1 \middle| \tau\right] - \mathrm{var}\left[\hat{\tau}_2 \middle| \tau\right]\right].$$

Identification

In the main text, we show how to write the RATE as a weighted average of the subgroup-specific treatment effects; however further identification steps are needed to express the RATE in terms of the observed data. In this section, we focus on identification of the subgroup-specific treatment effects within the observed sample. Identification proofs in this section closely follow those found in [1], with the exception that here we are estimating the subgroup-specific potential outcome means within the sample (i.e. conditional on $S = 1$) rather than estimating the subgroup-specific potential outcome means in an observed target population.

We require three assumptions

1. (Positivity of treatment assignment in the sample) For every $a \in \mathcal{A}$, $\mathbb{P}(A = a|X = x, S = 1) > 0$, where $f_X(x, S = 1) > 0$.
2. (Conditional exchangeability over treatment assignments in the sample) For every $a \in \mathcal{A}$, $\mathbb{E}[Y(a)|A = a, X, S = 1] = \mathbb{E}[Y(a)|X, S = 1]$.
3. (Consistency of treatment assignment in the sample) For every $a \in \mathcal{A}$, if $A = a$, then $Y = Y(a)$.

Using the three assumptions, we have that:

$$\begin{aligned} \mathbb{E}[Y(a)|X \in v_g, S = 1] &= \mathbb{E}\left[\mathbb{E}[Y(a)|X, S = 1] \middle| X \in v_g, S = 1\right] \\ &= \mathbb{E}\left[\mathbb{E}[Y(a)|A = a, X, S = 1] \middle| X \in v_g, S = 1\right] \\ &\qquad \textit{(positivity of treatment assignment in the sample)} \\ &\qquad \textit{(conditional exchangeability over treatment assignments given } X \textit{ and } S = 1\textit{)} \\ &= \mathbb{E}\left[\mathbb{E}[Y|A = a, X, S = 1] \middle| X \in v_g, S = 1\right] \\ &\qquad \textit{(consistency of treatment assignment)} \end{aligned}$$

Consequently, the subgroup-specific sample ATE can be expressed as:

$$\tau_g = \mathbb{E}\left[\mathbb{E}[Y|A = 1, X, S = 1] \middle| X \in v_g, S = 1\right] - \mathbb{E}\left[\mathbb{E}[Y|A = 0, X, S = 1] \middle| X \in v_g, S = 1\right]$$

We also show that the subgroup-specific sample ATE is algebraically equivalent to two inverse probability expressions. First, we have

$$\begin{aligned}
&\mathbb{E}\left[\mathbb{E}[Y|A=a,X,S=1]\middle|X\in v_g,S=1\right]\\
&=\frac{1}{\mathbb{P}(X\in v_g,S=1)}\,\mathbb{E}\left[\mathbb{1}(X\in v_g,S=1)\,\mathbb{E}[Y|A=a,X,S=1]\right]\\
&=\frac{1}{\mathbb{P}(X\in v_g,S=1)}\,\mathbb{E}\left[\mathbb{1}(X\in v_g,S=1)\frac{\mathbb{E}[Y|A=a,X,S=1]\,\mathbb{P}(A=a|X,S=1)}{\mathbb{P}(A=a|X,S=1)}\right]\\
&=\frac{1}{\mathbb{P}(X\in v_g,S=1)}\,\mathbb{E}\left[\mathbb{1}(X\in v_g,S=1)\frac{\mathbb{E}[\mathbb{1}(A=a)Y|X,S=1]}{\mathbb{P}(A=a|X,S=1)}\right]\\
&=\frac{1}{\mathbb{P}(X\in v_g,S=1)}\,\mathbb{E}\left[\mathbb{E}\left[\frac{\mathbb{1}(X\in v_g,S=1,A=a)Y}{\mathbb{P}(A=a|X,S=1)}\middle|X,S=1\right]\right]\\
&=\frac{1}{\mathbb{P}(X\in v_g,S=1)}\,\mathbb{E}\left[\frac{\mathbb{1}(X\in v_g,S=1,A=a)Y}{\mathbb{P}(A=a|X,S=1)}\right]\\
&=\frac{1}{\mathbb{P}(X\in v_g|S=1)\,\mathbb{P}(S=1)}\,\mathbb{E}\left[\frac{\mathbb{1}(X\in v_g,S=1,A=a)Y}{\mathbb{P}(A=a|X,S=1)}\right]
\end{aligned}$$

So, the subgroup-specific sample ATE is algebraically equivalent to:

$$\tau_g=\frac{1}{\mathbb{P}(X\in v_g|S=1)\,\mathbb{P}(S=1)}\left\{\mathbb{E}[w_1(X)Y]-\mathbb{E}[w_0(X)Y]\right\}\,,$$

where

$$w_a(X)=\frac{\mathbb{1}(X\in v_g,S=1,A=a)}{\mathbb{P}(A=a|X,S=1)}\,.$$

Second, note that

$$\begin{aligned}
\mathbb{E}\left[\frac{\mathbb{1}(X\in v_g,S=1,A=a)}{\mathbb{P}(A=a|X,S=1)}\right]&=\mathbb{E}\left[\mathbb{E}\left[\frac{\mathbb{1}(X\in v_g,S=1,A=a)}{\mathbb{P}(A=a|X,S=1)}\right]\middle|X,S=1\right]\\
&=\mathbb{E}\left[\frac{\mathbb{1}(X\in v_g,S=1)}{\mathbb{P}(A=a|X,S=1)}\,\mathbb{E}\left[\mathbb{1}(A=a)|X,S=1\right]\right]\\
&=\mathbb{P}(X\in v_g,S=1)\,.
\end{aligned}$$

Then, we can also express τ_g as

$$\tau_g=\left\{\mathbb{E}\left[w_1(X)\right]\right\}^{-1}\mathbb{E}[w_1(X)Y]-\left\{\mathbb{E}\left[w_0(X)\right]\right\}^{-1}\mathbb{E}[w_0(X)Y]\,.$$

Specifying subgroup representation for RATE estimators

Recall that we are interested in estimating τ using estimators of the form:

$$\tilde{\tau}=Q\hat{\tau}\,, \tag{1}$$

where Q is a matrix with entries $q_{ij}=\mathbb{P}(X\in v_j|S_i=1)$ and $\hat{\tau}$ is the column vector of unbiased estimators for τ. We handle each subgroup separately, focusing on $\tilde{\tau}_g=q_g^T\hat{\tau}$, where q_g^T is the gth row of Q. The risk function is:

$$\begin{aligned}
R(\tau_g,\tilde{\tau}_g)&=\mathbb{E}\left[(\tau_g-\tilde{\tau}_g)^2|\tau\right]\\
&=\mathrm{var}\left[\tau_g-\tilde{\tau}_g|\tau\right]+\left(\mathbb{E}\left[\tau_g-\tilde{\tau}_g|\tau\right]\right)^2\\
&=\mathrm{var}[q_g^T\hat{\tau}|\tau]+\left((e_g-q_g)^T\tau\right)^2\\
&=q_g^T\Sigma_{\hat{\tau}}q_g+(e_g-q_g)^T\tau\tau^T(e_g-q_g)\,,
\end{aligned}$$

where $\Sigma_{\hat{\tau}}$ is the covariance matrix for $\hat{\tau}$ keeping τ fixed. Different choices of q_g might be better under some values of τ and worse under others.

Average risk

The average risk, or Bayes risk, viewpoint seeks to choose an estimator that minimizes the average risk, given a specified prior distribution for τ.

$$\tilde{\tau}_g^{\text{Bayes}} = \arg\min_{\tilde{\tau}_g} \mathbb{E}_\tau[R(\tau_g, \tilde{\tau}_g)] \tag{2}$$

where $\tau \sim \pi$ and π is referred to as the prior distribution for τ. Rather than specifying π directly, we assume that π is exchangeable which means

$$\begin{aligned}\mathbb{E}_\tau[\tau] &= \mu_\tau \mathbf{1} \\ \Sigma_\tau &= \sigma_\tau^2(1-\rho)\mathbb{I} + \sigma_\tau^2 \rho \mathbf{1}\mathbf{1}^T\end{aligned}$$

Then taking the expectation over π, we can express the average risk as

$$\begin{aligned}\mathbb{E}_\tau[R(\tau_g, \tilde{\tau}_g)] &= q_g^T \Sigma_{\hat{\tau}} q_g + (e_g - q_g)^T \mathbb{E}_\tau\left[\tau\tau^T\right](e_g - q_g) \\ &= q_g^T \Sigma_{\hat{\tau}} q_g + (e_g - q_g)^T \Sigma_\tau (e_g - q_g) + \mu_\tau^2 \left(e_g^T \mathbf{1} - q_g^T \mathbf{1}\right)^2 \\ &= q_g^T \Sigma_{\hat{\tau}} q_g + (e_g - q_g)^T \Sigma_\tau (e_g - q_g) \\ &= q_g^T \Sigma_{\hat{\tau}} q_g + \phi^2 (e_g - q_g)^T (e_g - q_g)/2\end{aligned}$$

where $\phi^2 = 2\sigma_\tau^2(1-\rho) = \text{var}[\tau_i - \tau_j]$ for $i \neq j$.

To find q_g that minimizes the average risk, we want to solve:

$$\min_{q_g} \mathbb{E}_\tau[R(\tau_g, \tilde{\tau}_g)] \text{ subject to: } q_g^T \mathbf{1} = 1$$

To do this, we use the Lagrange multiplier method.

$$\nabla_{q_g, \lambda} \left\{ q_g^T \Sigma_{\hat{\tau}} q_g + \phi^2 (e_g - q_g)^T (e_g - q_g)/2 - \lambda(q_g^T \mathbf{1} - 1) \right\} = 0$$

First solving for q_g, we get

$$q_g = \Omega(\lambda \mathbf{1}/\phi^2 + e_g)\ ,$$

where $\Omega = \phi^2 \left(2\Sigma_{\hat{\tau}} + \phi^2 \mathbb{I}\right)^{-1}$. Applying the sum-to-one constraint, we have

$$\begin{aligned}&\mathbf{1}^T \Omega(\lambda \mathbf{1}/\phi^2 + e_g) = 1 \\ &\implies \lambda \mathbf{1}^T \Omega \mathbf{1}/\phi^2 + \mathbf{1}^T \Omega e_g = 1 \\ &\implies \lambda = \phi^2 (1 - \mathbf{1}^T \Omega e_g)(\mathbf{1}^T \Omega \mathbf{1})^{-1}\,.\end{aligned}$$

Plugging this back into the expression for q_g, we have

$$q_g = (1 - \mathbf{1}^T \Omega e_g)(\mathbf{1}^T \Omega \mathbf{1})^{-1} \Omega \mathbf{1} + \Omega e_g\,.$$

Note that when the entries of $\hat{\tau}$ are uncorrelated, i.e. $\Sigma_{\hat{\tau}}$ is a diagonal matrix with σ_g^2 as its gth diagonal entry, we can simplify the expression to

$$q_g = (1 - w_g) w \Big/ \left(\sum_{k=1}^G w_k \right) + w_g e_g$$

where $w_g = \frac{1/\sigma_g^2}{1/\sigma_g^2 + 2/\phi^2}$ and w is a column vector of $\{w_k\}_{k=1}^G$. Then the RATE estimator is

$$\tilde{\tau} = q_g^T \hat{\tau} = (1 - w_g)(\sum_{k=1}^G w_k \hat{\tau}_k)/(\sum_{k=1}^G w_k) + w_g \hat{\tau}_g\ .$$

If we further assume that the entries of τ are uncorrelated (i.e. $\rho = 0$), then $\phi^2/2 = \sigma_\tau^2$ and this is the same estimator that we get with a one-way random effects model.

Average risk with a shared set of weights

From the previous subsection, we have that for a general Q,

$$\mathbb{E}_\tau[R(\tau_g, \tilde{\tau}_g)] = q_g^T \Sigma_{\hat{\tau}} q_g + \phi^2 (e_g - q_g)^T (e_g - q_g)/2$$

where $\phi^2 = 2\sigma_\tau^2(1-\rho) = \text{var}[\tau_i - \tau_j]$ for $i \neq j$. In this subsection, we consider a specific form that will result in the same representation distributions for each subgroup, namely we require

$$q_g = w e_g + \frac{1-w}{G-1}(\mathbf{1} - e_g)\,,$$

where $w \in [0,1]$ and G is the total number of groups. Plugging in this expression for q_g, we have

$$\mathbb{E}_\tau[R(\tau_g, \tilde{\tau}_g)] = w^2\sigma_g^2 + 2\frac{w(1-w)}{G-1} e_g^T \Sigma_{\hat{\tau}}(\mathbf{1} - e_g) + \frac{(1-w)^2}{(G-1)^2}\left\{(\mathbf{1} - e_g)^T \Sigma_{\hat{\tau}}(\mathbf{1} - e_g) + \phi^2 G(G-1)/2\right\}\,,$$

where $\sigma_g^2 = e_g^T \Sigma_{\hat{\tau}} e_g = \text{var}(\hat{\tau}_g)$.

To ensure the same representation distributions for each subgroup, we aggregate the subgroup-specific objective functions by averaging over subgroups. The average risk, averaged over the subgroups, is

$$\begin{aligned} &G^{-1} \sum_{g \in \mathcal{G}} \mathbb{E}_\tau[R(\tau_g, \tilde{\tau}_g)] \\ &= \left[w^2 (G-1)^2 \bar{\sigma^2} + 2w(1-w)(G-1)V_1 + (1-w)^2\left\{V_2 + \phi^2 G(G-1)/2\right\}\right]/(G-1)^2\,, \end{aligned}$$

where $\bar{\sigma^2} = G^{-1}\sum_{g\in\mathcal{G}} \sigma_g^2$, $V_1 = G^{-1}\sum_{g\in\mathcal{G}} e_g^T \Sigma_{\hat{\tau}}(\mathbf{1} - e_g)$, and $V_2 = G^{-1}\sum_{g\in\mathcal{G}}(\mathbf{1} - e_g)^T \Sigma_{\hat{\tau}}(\mathbf{1} - e_g)$. This joint objective function is quadratic in w with a leading coefficient of

$$\left[(G-1)^2\bar{\sigma^2} - 2(G-1)V_1 + V_2 + \phi^2 G(G-1)/2\right]/(G-1)^2\,.$$

When subgroup effect estimates are uncorrelated, $V_1 = 0$ and $V_2 > 0$, which means the leading coefficient is definitely positive. However, it is unclear whether this coefficient is positive in general. As sample size increases terms that depend on the covariance matrix of the subgroup-specific effect estimates $(\bar{\sigma^2}, V_1, V_2)$ will go to 0. So with sufficient sample size, as long as $\phi^2 > 0$, this leading coefficient should be positive, which would mean the objective function is convex. In general, researchers should check this regularity condition, that is, that this quantity is greater than 0.

To find the optimal w, we set the derivative equal to 0 and solve for w. We find

$$w = \frac{1}{1+\gamma}$$

where $\gamma = \frac{\bar{\sigma^2}(G-1) - V_1}{\phi^2 G/2 + V_2/(G-1) - V_1}$. Note that we need $\gamma \geq 0$ in order for $w \in [0,1]$. Again, $\gamma > 0$ when subgroup effect estimates are uncorrelated; however, this might not hold in general and should be verified. We confirmed that these pathological cases did not occur in any of our simulations.

Large sample properties of RATE

For both versions of the RATE estimators, we have that $\lim_{n\to\infty} q_g = e_g$. If we assume that the subgroup-specific estimators are asymptotically normal and consistent for the true parameters (i.e. $\hat{\tau}_g \xrightarrow{d} N(\tau_g, \sigma_g)$), then by Slutsky's theorem we have that the RATE estimator has the same asymptotic distribution $\tilde{\tau}_g \xrightarrow{d} N(\tau_g, \sigma_g)$.

Additional simulation results

Summary statistics for the simulations shown in Figure 3 in the main text.

Group	Estimator	Min.	25%	50%	75%	Max.
Group 1	SATE	0.111	0.136	0.196	0.293	0.850
	Stratification	0.119	0.132	0.134	0.137	0.146
	Interaction	0.119	0.131	0.134	0.137	0.146
	Random effects	0.116	0.130	0.135	0.140	0.157
	RATE-shared	0.112	0.127	0.152	0.202	0.516
	RATE-opt (1.5)	0.118	0.130	0.133	0.136	0.146
	RATE-opt (1)	0.118	0.129	0.132	0.137	0.183
	RATE-opt (0.75)	0.117	0.130	0.132	0.135	0.155
Group 2	SATE	0.116	0.285	0.568	0.915	2.618
	Stratification	0.282	0.299	0.306	0.312	0.336
	Interaction	0.274	0.293	0.299	0.305	0.332
	Random effects	0.182	0.271	0.303	0.329	0.403
	RATE-shared	0.240	0.260	0.273	0.298	0.466
	RATE-opt (1.5)	0.265	0.281	0.287	0.294	0.324
	RATE-opt (1)	0.236	0.257	0.271	0.302	0.503
	RATE-opt (0.75)	0.252	0.271	0.278	0.291	0.385
Group 3	SATE	0.113	0.318	0.601	0.941	2.483
	Stratification	0.349	0.373	0.380	0.387	0.415
	Interaction	0.336	0.360	0.367	0.375	0.402
	Random effects	0.183	0.322	0.362	0.416	0.520
	RATE-shared	0.291	0.316	0.328	0.346	0.495
	RATE-opt (1.5)	0.313	0.338	0.345	0.355	0.393
	RATE-opt (1)	0.267	0.294	0.315	0.363	0.666
	RATE-opt (0.75)	0.291	0.316	0.329	0.346	0.492

Table S1: Summary statistics for RMSE distribution for each estimator for each group. Min. = minimum; Max. = maximum; and 25%, 50%, and 75% are the 25th, 50th, and 75th percentiles, respectively. This simulation is based on 500 draws of 3 subgroup effects from a standard normal distribution scaled so that $\phi^2 = 1$.

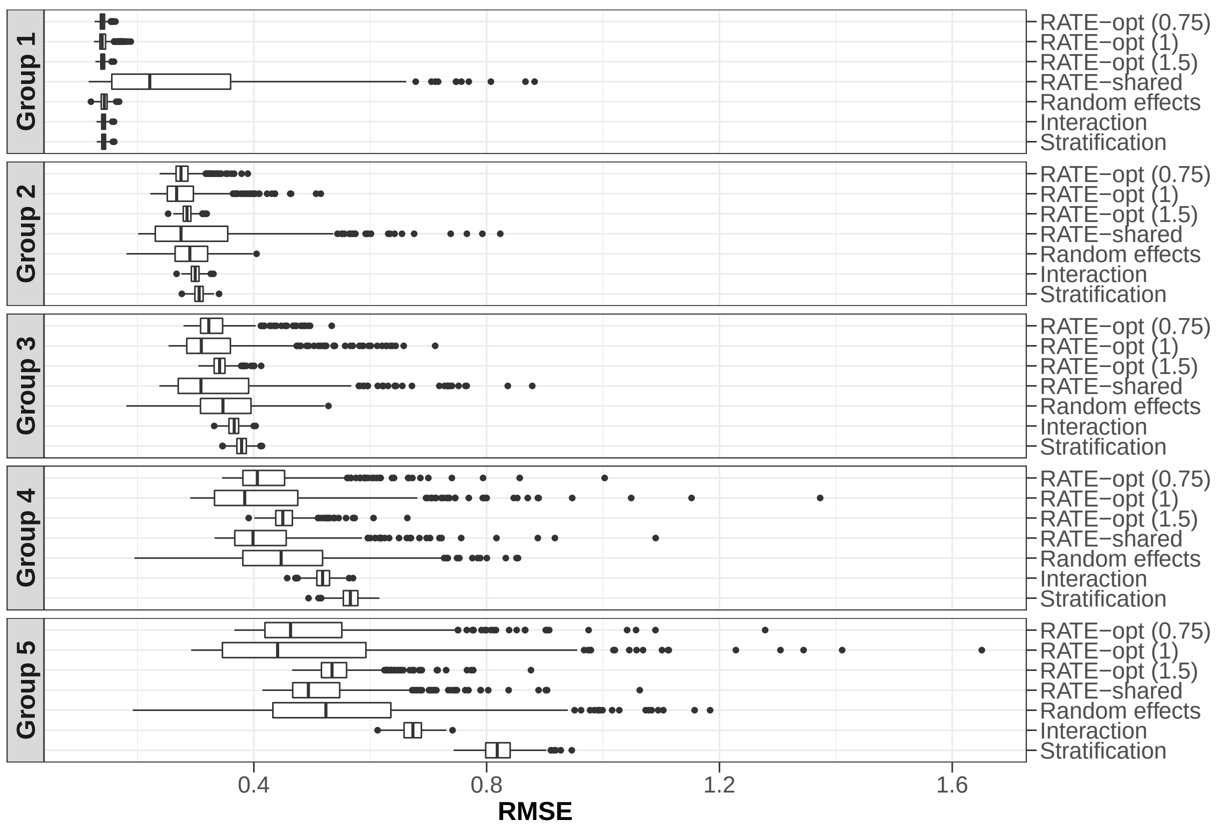

Figure S1: Box plots of RMSE of estimators of subgroup-specific treatment effects; based on 500 draws of 5 subgroup effects from a standard normal distribution scaled so that $\phi^2 = 1$.

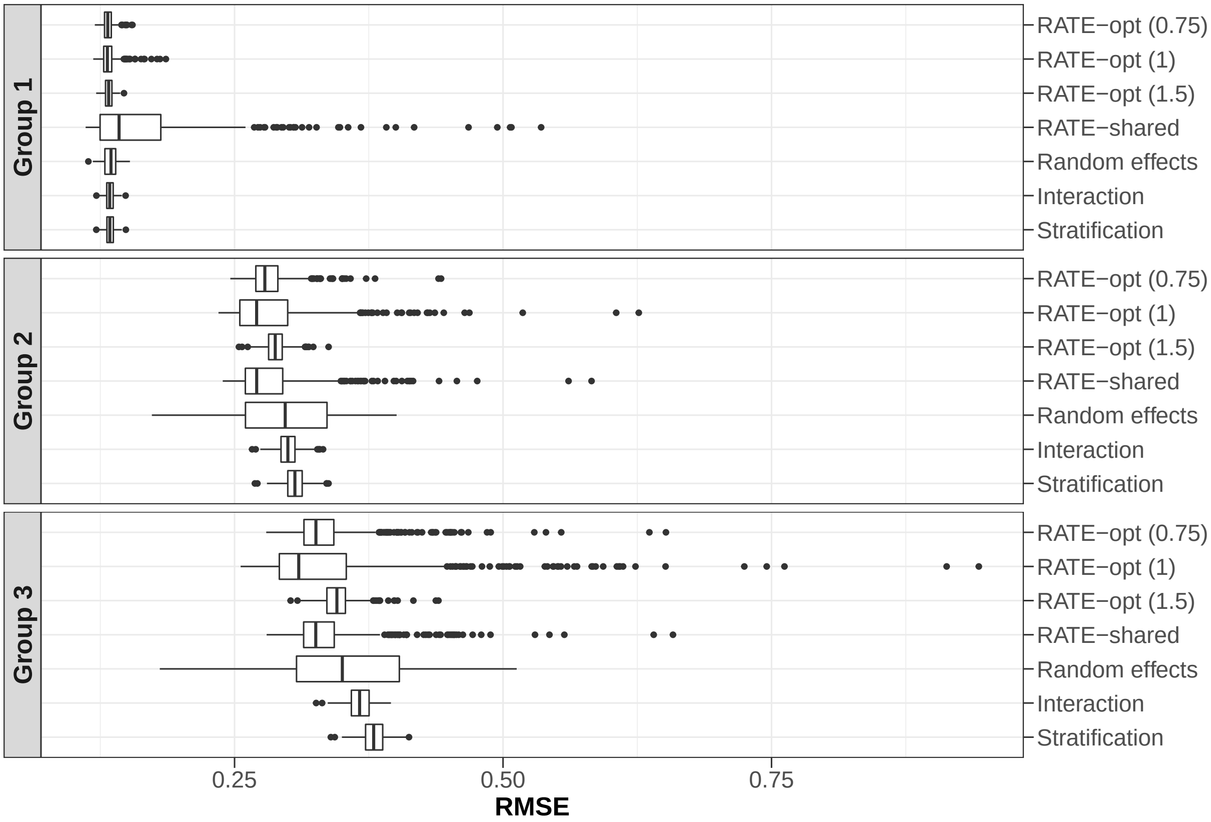

Figure S2: Box plots of RMSE of estimators of subgroup-specific treatment effects; based on 500 draws of 3 subgroup effects from a Gamma(3,3) distribution scaled so that $\phi^2 = 1$.

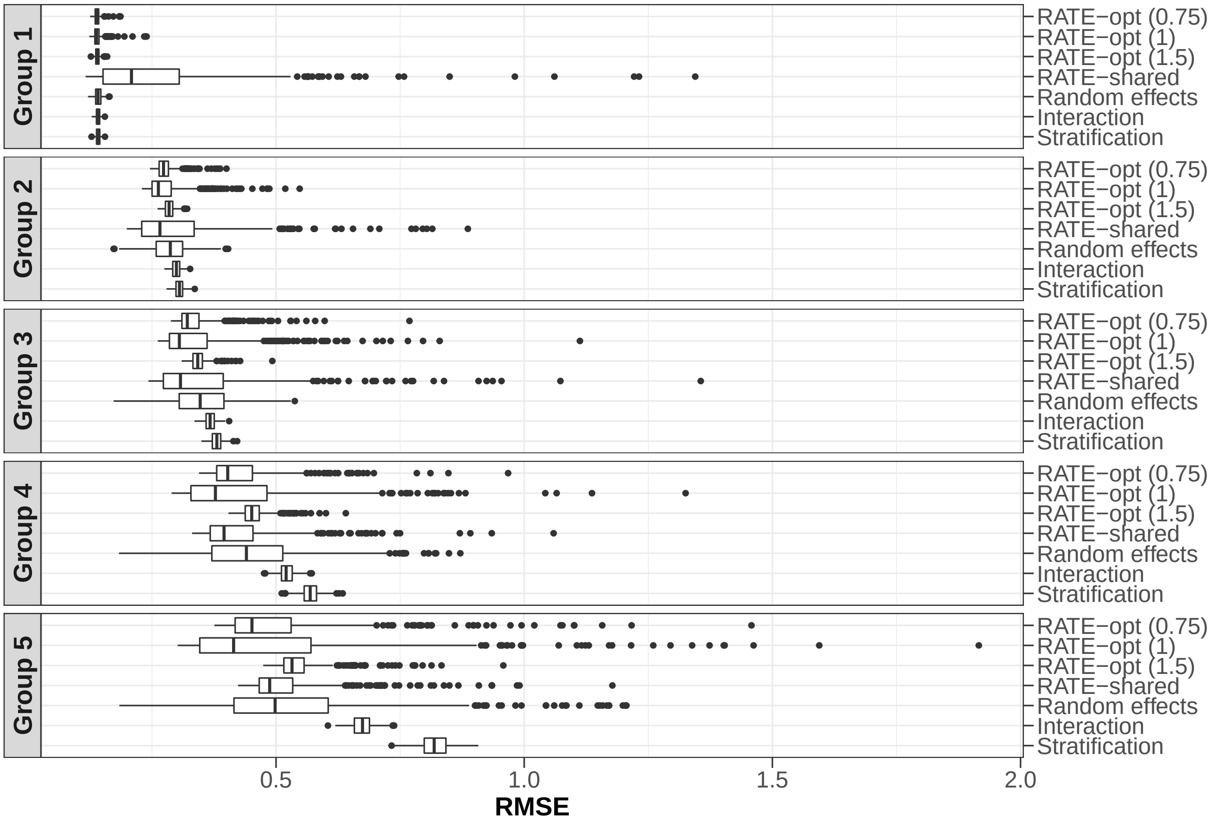

Figure S3: Box plots of RMSE of estimators of subgroup-specific treatment effects; based on 500 draws of 5 subgroup effects from a Gamma(3,3) distribution scaled so that $\phi^2 = 1$.

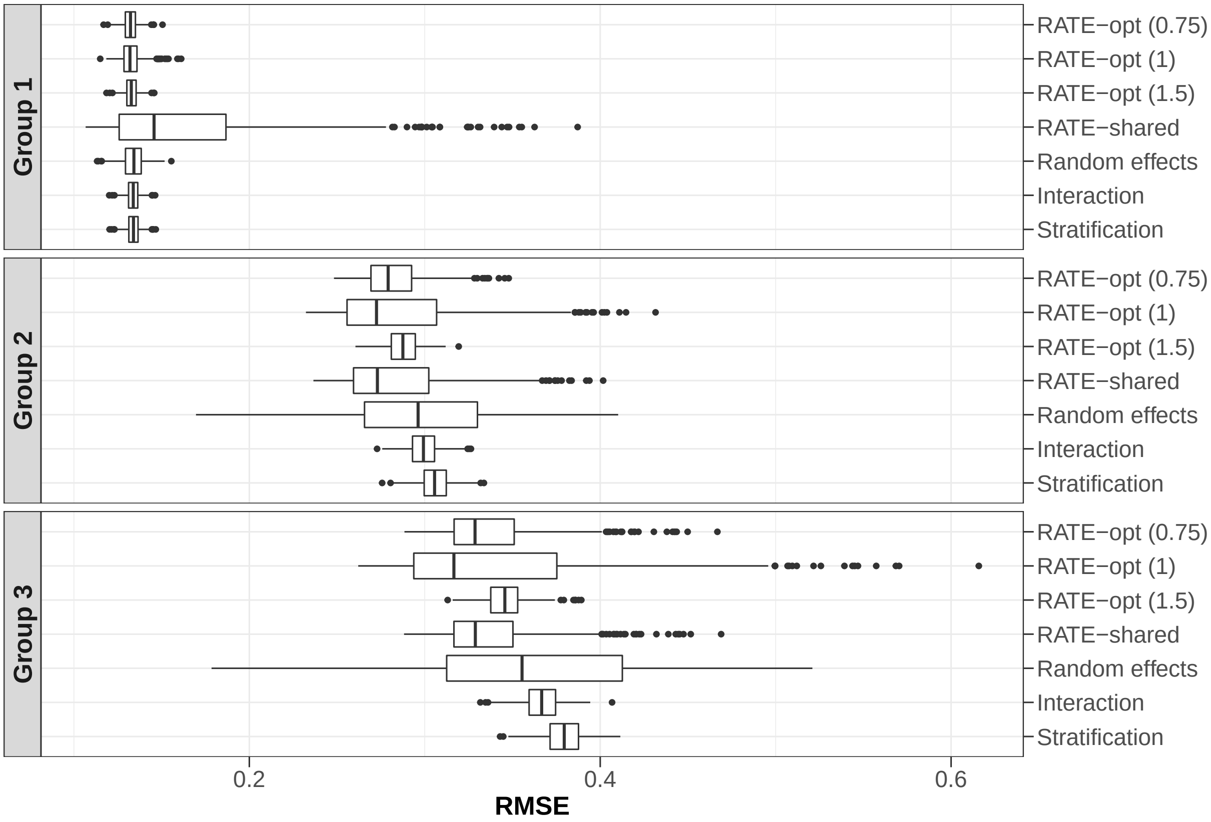

Figure S4: Box plots of RMSE of estimators of subgroup-specific treatment effects; based on 500 draws of 3 subgroup effects from a mixture of two normal distributions—N(0.5, 1) with probability 0.8 and N(-3, .5) with probability 0.2—scaled so that $\phi^2 = 1$.

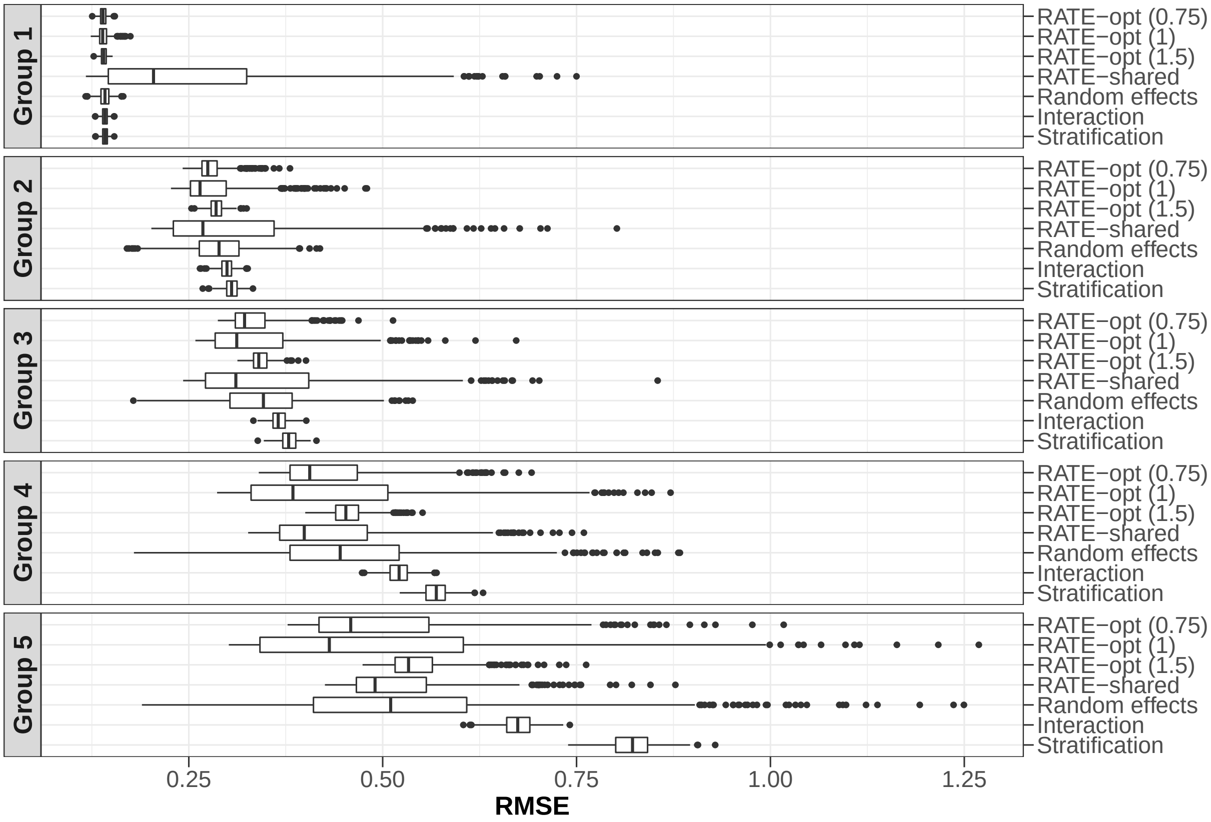

Figure S5: Box plots of RMSE of estimators of subgroup-specific treatment effects; based on 500 draws of 5 subgroup effects from a mixture of two normal distributions—N(0.5, 1) with probability 0.8 and N(-3, .5) with probability 0.2—scaled so that $\phi^2 = 1$.

Case study detail

The full representation matrix calculated for the case study was:

$$Q = \begin{array}{c} \\ NVG \\ VG \\ NVB \\ VB \end{array} \begin{array}{c} \begin{array}{cccc} NVG & VG & NVB & VB \end{array} \\ \begin{pmatrix} 0.724 & 0.087 & 0.105 & 0.083 \\ 0.131 & 0.633 & 0.132 & 0.104 \\ 0.101 & 0.085 & 0.732 & 0.081 \\ 0.138 & 0.116 & 0.140 & 0.605 \end{pmatrix} \end{array}$$

References

1. Robertson, S. E., Steingrimsson, J. A., Joyce, N. R., Stuart, E. A. & Dahabreh, I. J. Estimating subgroup effects in generalizability and transportability analyses. *American Journal of Epidemiology* (2022).
